# Development of and Experiences With an Informational Website on Early Labor: Qualitative User Involvement Study

**DOI:** 10.2196/28698

**Published:** 2021-09-27

**Authors:** Enid Leren Myhre, Lisa Garnweidner-Holme, Bente Dahl, Marte Myhre Reigstad, Mirjam Lukasse

**Affiliations:** 1 Centre for Women’s, Family and Child Health Faculty of Health Sciences University of South-Eastern Norway Kongsberg Norway; 2 Faculty of Health Sciences Department of Nursing and Health Promotion Oslo Metropolitan University Oslo Norway; 3 Norwegian Research Centre for Women's Health Department of Obstetrics and Gynecology Oslo University Hospital Oslo Norway

**Keywords:** early labor, latent phase, think aloud, usability, website, labor, pregnancy, user-friendliness, eHealth, user satisfaction

## Abstract

**Background:**

The period of regular contractions before 4 cm of cervical dilatation is often referred to as the *latent phase* or *early labor*. Women find it challenging to prepare for and cope with this phase of labor, and easily accessed web-based information from reliable sources may be useful in this preparation.

**Objective:**

The aim of this study is to describe the development of a Norwegian website, Latens.no, for people seeking information on early labor and to explore users’ experiences with the website to increase its user-friendliness.

**Methods:**

We developed a website using an iterative process involving a multidisciplinary research team, health personnel, users, a graphic designer, and an expert in software development. We explored the website’s user-friendliness using semistructured individual interviews and the think-aloud method. All interviews were audio recorded and transcribed. We then analyzed the participants’ feedback on the website.

**Results:**

Participants included women who had recently given birth to their first baby (n=2), women who were pregnant with their first baby (n=4), and their partners (n=2). Results from participants’ experiences completing tasks included positive feedback related to the content of Latens.no, positive feedback related to the website’s design, and suggestions for improvement. Participants wanted to find information on early labor on the internet. Moreover, they found the information on the website relevant, trustworthy, and easy to read, and the design was attractive and easy to use. Overall, the participants performed the tasks easily, with few clicks and minimal effort.

**Conclusions:**

The think-aloud method, while performing tasks, allowed for detailed feedback. The participants confirmed the user-friendliness of the website but at the same time provided information enabling improvement. We expect that changes made based on this user-centered design study will further increase the usability and acceptability of Latens.no.

## Introduction

### Background

The period of regular contractions before 4 cm of cervical dilatation is often referred to as the *latent phase* or *early labor* [1]. This period can be considered a time of conflict between women’s perceived need to be cared for and midwives’ determination to prevent them from coming to the hospital earlier than necessary. We favor the term *early labor*, as women do not consider labor to consist of different phases [2], and it captures the fact that this phase is actually a part of the labor process.

The duration of early labor differs tremendously: although it can be short for some women, for others, it may continue for hours or even days [3-5]. A lack of satisfaction with care provided in early labor before hospital admission has been reported in prior studies [6,7]. A metasynthesis of experiences from first-time mothers in early labor suggests that women’s needs are often not adequately met [7]. The authors describe a mismatch between women’s expectations and experiences; in particular, women expressed uncertainty about interpreting signs and symptoms when determining the need for admission [7]. In a systematic review, Beake et al [6] concluded that women, labor companions, and even health professionals found early labor difficult to manage well.

Nevertheless, the National Institute for Health and Care Excellence guidelines recommend that women seeking advice when not in established labor should be encouraged to remain at or return home, unless doing so would lead to severe distress or a significant risk of birth without a midwife present [8]. Although some midwives offer home births in Norway, giving birth in a hospital is the norm. Clinical practice recommendations in Norway state that women in early but not active labor should generally not be admitted to the hospital [9]. This is in accordance with research investigating outcome differences between women presenting at the hospital in early versus active labor [10]. Results from several large studies suggest that hospital admission in early labor is associated with an increased risk of medical interventions, including electronic fetal monitoring [11], epidural analgesia [12,13], oxytocin stimulation [12,13], and cesarean section [12,13].

The National Institute for Health and Care Excellence guidelines recommend educating first-time mothers about early labor [8]. A randomized controlled trial found that women receiving structured antenatal training programs arrived at the maternity ward in active labor more often and used less epidural analgesia than those receiving routine care [14]. Furthermore, a systematic review investigating maternal confidence for physiologic birth suggests that women desire information during pregnancy and want to use that information to participate in care decisions [15]. Altogether, evidence suggests that easy access to relevant and reliable information could be a way of supporting and empowering women to cope with early labor. In addition, educating women’s labor companions is recommended to enable them to feel more confident, and thus, provide better support at home [6].

The information needs of first-time mothers during pregnancy seem to be increasing [16,17]. Although knowledge is easily accessible because of advances in information technology, it is important for health professionals to consider the amount and type of information they communicate [16,18]. The increasing use of web-based information requires further research [6]. A systematic review of research on health information needs, sources of information, and barriers to accessing health information among pregnant women found that although several studies have examined the information needs of pregnant women, further qualitative research is recommended to explore pregnant women’s perceptions of and satisfaction with the use of health information sources [19].

### The PreCare Study

This study was part of the PreCare study (preadmission early labor care: an electronic educational intervention to improve information flow in early labor care and women’s preadmission early labor experience). The overall aim of the PreCare study is to develop a web-based educational resource for women in early labor and to test its effectiveness on women’s experience of early labor.

The PreCare study began by exploring women’s experience with existing information and their knowledge needs in preadmission early labor. The findings suggest that easy access to a suitable amount of trustworthy information at the appropriate time has a positive impact on reassuring women in early labor [20]. Participants did not necessarily need large amounts of information but wished for useful, readily accessible information about the *usual stuff* that was easy to comprehend and relate to [20]. The results from this study informed the initial content of the website Latens.no.

In subsequent studies, we will investigate whether the website improves women’s knowledge of early labor, experience in early labor, and explore whether the use of the website affects clinical birth outcomes related to giving birth.

The aim of this study is two-fold: the first objective is to describe the development of Latens.no, whereas the second is to explore users’ experiences with the website.

## Methods

### Development of Latens.no

**Figure 1:**
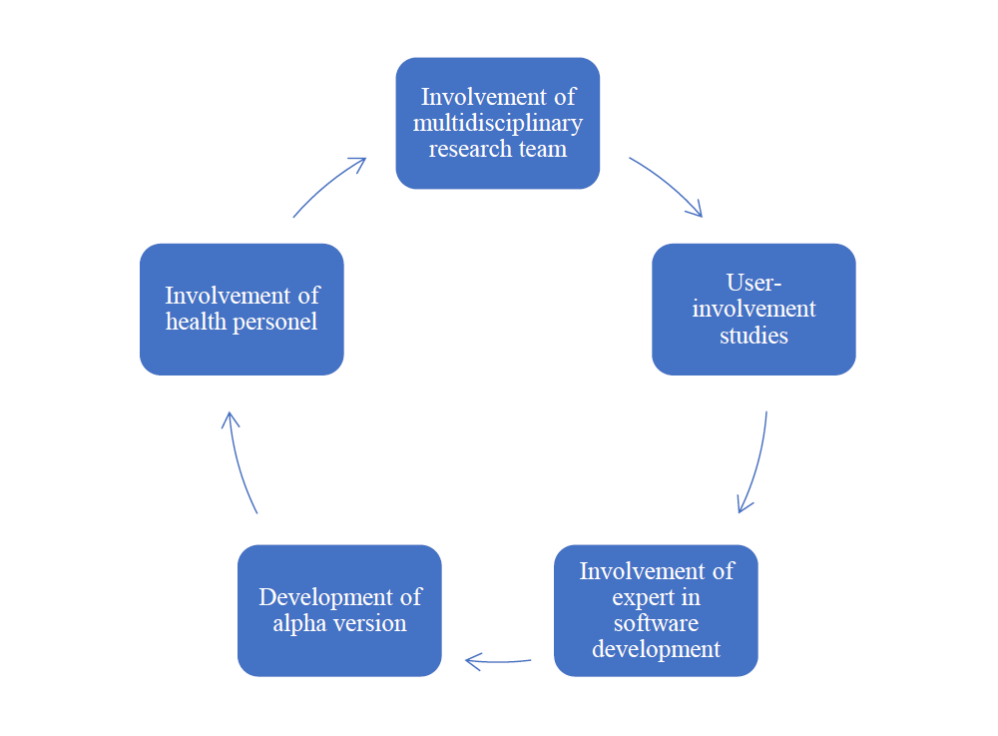
Illustration of the iterative process of developing the website.

To secure content quality and increase usability, the website was developed through an iterative process involving a multidisciplinary research team consisting of 3 midwives, a gynecologist, health personnel, people in the target group, and an expert in software development. In addition, the graphic designer provided custom-made illustrations. Figure 1 illustrates the website development process.

#### Multidisciplinary Research Team and Health Personnel Involvement

We created an alpha version of the website with input from the first user-involvement study and discussions among the multidisciplinary research team. The alpha version of Latens.no consisted of a front page with an image and a description of the content, in addition to a top banner with a logo, a menu, and a search option (Multimedia Appendices 1 and 2). From the menu or search option, users could access 10 different pages with relevant content about early labor. All pages had illustrative images, text, and an option to click on subheadings if the user wanted more detailed explanations. The pages also had hyperlinks, both internal hyperlinks linked to related content on Latens.no, and external hyperlinks linking to relevant information, such as information from the World Health Organization. In addition, some pages contained informative images or short videos. The website was assessed by the first author (ELM) with the help of the Suitability Assessment of Materials (SAM) for evaluating health-related information for adults. The SAM provides a validated method for evaluating written health-related education material in terms of the categories and factors known to enhance people’s understanding of printed materials [21]. Each factor (content, literacy demand, graphic illustrations and lists, layout and typography, learning stimulation and motivation, and cultural appropriateness) was assessed and deemed either superior, adequate, or not suitable. Changes to Latens.no were made accordingly.

In addition, we asked midwives who work on a daily basis with women in early labor for input by posting the alpha version in a closed Norwegian Facebook group of 76 members (all midwives or nurses and all Norwegian speaking). We requested honest feedback on both content and design. Responses were mainly *likes* or comments such as *this is very good*. However, a few members of the Facebook group provided constructive feedback. In addition, the first author observed that 3 midwives provided oral feedback while they systematically looked through the entire website. Minor revisions were then made based on feedback from the involvement of these health personnel. Changes were mainly aimed at language, such as removing small words that are often associated with something negative (eg, *but* and *unfortunately*) and adding improved explanations for some of the most-used words and phrases.

#### User Involvement

Initially, we asked the participants to describe their general preferences related to information websites on pregnancy and birth, in terms of both content and design. To assess their experiences using Latens.no, end users then tested an alpha version of Latens.no using the *think aloud* method [22]. By instructing participants to think aloud as they used the website, we aimed to collect participants’ mental processing while carrying out a task, to discover potential usability problems. We asked the participants to complete 10 tasks with the help of the website while continuously thinking aloud. The tasks consisted of hypothetical situations that could occur in early labor (Multimedia Appendix 3). The participants were not given access to the website before the testing started. The testing was completed after a brief follow-up interview to clarify the participants’ thinking and possible misunderstandings. All interviews were audio recorded and transcribed verbatim.

### Recruitment

Participants were purposely recruited at well-baby clinics in South-Eastern Norway from February to June 2020. Midwives at well-baby clinics invited eligible women and their partners to participate. Owing to the midwives’ heavier workload from the COVID-19 pandemic and to limited response rates, we proceeded to recruit via snowball sampling. Initially, women who had recently given birth to their first baby were recruited, along with their partners. However, it soon became apparent that these participants had considerable knowledge on the subject of early labor. This raised concerns about whether they would be critical enough in their feedback on Latens.no. As a result, we continued the testing with the help of women who were pregnant with their first baby and their partners.

### Ethical Considerations

The study was conducted in accordance with the World Medical Association Declaration of Helsinki [23]. Participants were advised that participation was voluntary and that they could withdraw from the study at any time without giving reasons. They were provided with information, given time to consider whether they wanted to participate, and informed consent was obtained from all participants. Approval for the study was granted by the Norwegian Centre for Research Data (NSD: 228701).

### Data Analysis

We analyzed the data using thematic analysis, following the study by Braun and Clarke [24,25]. In the process of analysis, we did not look for anything beyond what our participants said, instead focusing on identifying themes at a semantic level. We followed Braun and Clarke’s five phases of analysis throughout the process. First, after familiarizing ourselves with the data by repeated reading of each informant’s transcripts, the first author (ELM) and last author (LGH) generated initial codes. As we coded for as many potential themes as possible, not all initial codes were related to the usability of health information websites. Next, all codes were collated into potential subthemes and main themes before we checked how the themes worked in relation to the initial codes and the entire data set. NVivo was used to identify and organize the codes and themes (version 12; QSR International). We then looked at the overall story of the analyses, searching for themes relevant to the usability of the website. Finally, we agreed on the clear definitions and names of the subthemes and themes. All 5 authors were involved in the end stage of the analysis and discussed the themes until we reached a consensus. We carried out a thematic analysis only on data relevant to how our informants experienced the site.

## Results

### Overview

A total of 8 participants verbalized their experiences and completed tasks on Latens.no and in think-aloud interviews. The characteristics of the participants are presented in Table 1.

In general, participants expressed a need to find information on early labor on the internet. They talked about the information they had looked for but did not find. In addition, they described the types of information they would appreciate. Some asked for concrete information: for example, about practice and false labor contractions, the duration of early labor, and what to do when contractions start. A few described how they wanted to be reassured that what they were experiencing was normal. The analysis of participants’ feedback on Latens.no resulted in three main themes informed by nine subthemes (Textbox 1): positive feedback related to the content on Latens.no, positive feedback related to design, and suggestions for improvement.

**Table 1:** Characteristics of participants (N=8).

Characteristics			Participants
**Age (years), n (%)**			
	26-30	2 (25)	
	31-37	5 (63)	
	38-41	1 (13)	
**In a partnership, n (%)**			
	Yes	7 (88)	
	No	1 (13)	
**Reason to have knowledge about early labor, n (%)**			
	Pregnant	4 (50)	
	Had recently given birth	2 (25)	
	Partner to pregnant woman	1 (13)	
	Partner to woman who had recently given birth	1 (13)	
**Level of education^a^, n (%)**			
	Upper secondary, final year	1 (13)	
	Postsecondary nontertiary education	1 (13)	
	First stage of tertiary education, undergraduate level	4 (50)	
	First stage of tertiary education, graduate level	1 (13)	
	Second stage of tertiary education (postgraduate education)	1 (13)	
**Participation of partner in early labor, n (%)**			
	Partner present	1 (13)	
	Partner present in some part of early labor	2 (25)	
	Had not made plans for this	3 (38)	
	Planned for partner to be present	2 (25)	
**Device used to access Latens.no, n (%)**			
	Mobile phone	6 (75)	
	PC	2 (25)	

^a^The Norwegian Standard Classification of Education (NUS2000).

**Main themes and subthemes.**

**Positive feedback related to content on Latens.no**

- The information on Latens.no is relevant
- Latens.no is perceived as trustworthy
- The text on Latens.no is easy to read

**Positive feedback related to design on Latens.no**

- Colors and images make Latens.no attractive
- Latens.no is clearly structured and easy to use

**Suggestions for improvement on Latens.no**

- Requests and suggestions for improvement of features
- Requests and suggestions for improved readability
- Negative feedback related to inconsistent layout
- Negative feedback related to images

### Positive Feedback on Content

Participants expressed that the information on Latens.no was relevant to them. Some explained how their own experience and knowledge made the information on the webpage more relevant. One participant appreciated that Latens.no contained all necessary information in one place, reducing the need to spend time using Google. This view was echoed by another participant:

I think the pages with the drawings and the pictures and the information, it feels like exactly what I would have needed.

Interview 1

Trustworthiness was of great importance to our participants when searching for information related to early labor. For instance, 1 participant expressed that she would prefer to receive web-based information about pregnancy and childbirth from health professionals. However, the majority of our participants assessed trustworthiness on other criteria. One participant emphasized that information needed to be up to date (although she did not indicate how she assessed this information). Another commented that typos and misspellings generally ruined the credibility of a website for her. A third noted that websites that looked unprofessional gave her a bad impression. Commenting on the content of Latens.no, one participant explained why she perceived it as trustworthy:

Professional, I think. Yeah, I can see that it is made by people who know what they are talking about. There are few typos and things like that.

Interview 3

Most of the participants remarked that Latens.no was easy to read. Several said that the fact that it had few unnecessary elements improved readability. One participant used the word *clean* and stated that this made the website comprehensible. Other statements related to the language used on the website. One participant commented that because the text was short, concise, and easy to read, it was easy to locate information. Another participant remarked on how she would return to this website, as opposed to many other websites, when in labor. She said she found the information interesting, and although she could probably find the information other places, Latens.no made it easy to understand. Furthermore, some participants pointed out that the website’s use of *layman’s terms* made it understandable. One participant reported the following:

I found it easy to understand. Not everyone are nurses and medical students and who knows what. So, it was kind of easy to understand it. At the same time, it wasn’t written in kindergarten language. So, it was really easy to understand.

Interview 7

### Positive Feedback on Design

Many participants provided positive feedback related to the design of Latens.no. When asked about images, one individual answered that he did not notice the images but still enjoyed the design. Several others commented on how they appreciated the images and said they found a suitable amount on Latens.no. Furthermore, several participants remarked that Latens.no had a pleasant design. One participant argued that its clean design was attractive, whereas others proposed that its feminine design was suitable for the target group. Several participants also expressed that the use of colors in Latens.no was appealing. For example, one participant reported the following:

It has a pleasant design, I must say. Yes. It has pleasant colors that suit—I think it really suits the subject.

Interview 4

As with other websites, participants valued that Latens.no was easy to navigate; they found it to be clearly structured and easy to use. Some noted that the headings and subheadings were clear, which made it easy to navigate. A few participants stated that the search engine was easy to use, and one explained that she liked that the content was categorized. Others simply said that information was easily obtained. One participant said she found it encouraging and reassuring that Latens.no was so easy to navigate. When asked to suggest modifications, one participant reported the following:

I do not think it should be modified too much. Because it can easily become too much. You have a menu. And you have your headings and subheadings—or, like, points—and then explanations beneath the points.

Interview 6

### Suggestions for Improvement

Despite the overall positive response, there were suggestions on how to improve Latens.no. Some recommendations were related to improvements in the design features. One participant mentioned that the menu was plain and suggested the use of bullet points or colors, whereas others argued that it was not clear that the subheadings were clickable. A total of 2 participants remarked that they misunderstood the *contact information* section, with 1 participant suggesting that it was added to the menu. A total of 2 participants experienced difficulties in using the search engine. Some participants expressed a need to receive information from a *partner perspective* on what to do, what to expect, and what is happening, and another expressed a desire for separate information for women and partners:

Yes, you could separate the practical information, like you have one with practical information for the woman, and one with practical information for the guys, or whoever it is. Separate them, maybe? Or “What can I do to feel better at home?”—I reckon that one is meant for the one who is giving birth. So maybe it could be clear instructions on what you can do as a father, or, yes.

Interview 2

Other suggestions were related to readability. Some participants suggested that the font size was too small. Another commented that she noticed some spelling mistakes. One participant suggested that the front page could be *bigge*r, with more images, and 2 participants indicated that it contained too much text:

It was a bit like, when you first enter the website, there is a lot of information, right there. It is a bit, it looks a bit much.

Interview 7

The participants gave negative feedback related to the images on Latens.no. One participant noted that the text placed across images could be hard to read, and another felt that the images might be too large. Another suggested the use of more images:

I am a fan of pictures. So, there could probably be more pictures. Because, yes, for us that are visual.

Interview 3

Finally, the website’s inconsistent layout was critiqued. One participant pointed out the use of different font sizes and inconsistent formatting of subheadings:

A thing that bothers me a little bit, kind of like, that it has different text fonts.

Interview 8

### Observations During Tasks

The interviews lasted an average of 14 minutes, with a range of 9-23 min; in most of this time, participants used the website. The participants continuously verbalized how they navigated the website while performing the tasks they were given. Therefore, a part of our empirical material consists of descriptions of what they were looking at and how they were navigating the site. All participants referred to the menu many times during the test. There are numerous quotes similar to this example (the participant was asked to find out what to bring to the hospital):

Let me see. I enter the menu again. And then I look at the list of links. Let me see. And then I click the link called “practical information,” and there I see a packing list. So I found it there.

Interview 4

The search option was also frequently mentioned. Many found what they were looking for by performing simple searches. As noted earlier, however, 2 participants experienced difficulties in searching:

Then I simply search “blood on toilet paper.” I am thinking that might be wise. No, I don’t know if it will perform the search? Or am I doing something wrong? I don’t think it will perform the search.

Interview 5

Overall, participants performed the tasks easily, with few clicks and minimal effort, and our empirical material contains many quotes, such as the following:

It was very easy to find, really.

Interview 3

### Adjustments Informed by Study Findings

Latens.no was refined based on feedback from the interviews and think-aloud results (Multimedia Appendices 4-6). The search option was optimized, minor alterations were made to the menu, and small adjustments were made on the front page. We increased the font size to improve readability, and an additional round of proofreading was conducted to eliminate any spelling mistakes. We adjusted the subheadings, removed text across images, and made minor revisions to the image sizes. Although we received some feedback related to the number of images, we decided to keep the original number of images because several participants stated that the website’s simplicity improved its readability. Finally, as Latens.no already contained separate information for women and partners, we made no adjustments related to this.

## Discussion

### Principal Findings and Comparison With Prior Work

The purpose of this study was to describe the development of a website with information on early labor and to explore the experiences of women and their partners using the website. All of the participants wanted to find information on early labor using the internet; moreover, they found the information on Latens.no relevant, trustworthy, and easy to read, and the design was attractive and easy to use.

The finding that all our participants had a desire to find information on early labor using the internet corresponds with findings reported in the first article in the PreCare study [20]; it also broadly supports the findings of other studies in this area [16,17,19,26]. As mentioned in the introduction, there appears to be an increasing need among first-time mothers for information about early labor [16,17]. The internet is an attractive option for obtaining pregnancy-related information because of its convenience, availability, and anonymity [27]. In a systematic review, Ghiasi et al [19] identified digital media as one of the most common health information sources for women during pregnancy. A descriptive cross-sectional study on the internet behavior of women who were pregnant or trying to conceive found that 95.6% of their participants used the internet as an information source before or during their pregnancy [26]. In line with previous studies, our participants searched the internet for both concrete information and reassurance that what they were experiencing was normal [26,28-30]. Finding various kinds of information related to contractions was frequently mentioned by our participants. This was also among the main topics that women searched for in other studies [29]. However, Bjelke et al [28] found that the primary reason pregnant women search the internet is to find information and read about people in the same situation, which is consistent with our finding that the women in our study wanted reassurance that what they were experiencing was normal. As noted earlier, we recruited both women who had recently given birth to their first baby and their partners, but we ultimately focused on women who were pregnant with their first baby and their partners. As such, many of our participants were first-time mothers and their partners. It is recommended that first-time mothers receive education about early labor through antenatal care [8]. However, they might need additional information, and previous studies on internet use among pregnant women indicate that first-time mothers are more likely to seek advice than multiparous women [29,30].

A meta-analytic review of tailored print health behavior change interventions demonstrated that personally relevant websites were more effective [31]. This corresponds to our study, in which a key finding was that participants found information on Latens.no useful and relevant. This may be because of user involvement from the beginning of the website’s development. In their systematic review on the usability and effectiveness of mobile health technology, Overdijkink et al [32] recommended that the development of new health technology should be done together with the target group. Another reason the content was perceived as relevant could be that we had involved health personnel who work clinically with women in early labor. This finding is encouraging and shows that Latens.no has the potential to be an effective web-based educational resource for women in early labor.

Our participants were concerned about trustworthiness, and indeed they generally found information on Latens.no to be trustworthy. In previous web-based studies, only approximately half of the pregnant women trusted the information they found [30]. In contrast, in a systematic review of internet use by pregnant women seeking information, the authors suggest that the majority of pregnant women with higher education perceive web-based health information to be trustworthy [29]. As the overall level of education among our participants was high, this may explain why our participants found information on Latens.no trustworthy.

The overall high level of education might also explain another important finding: our participants found the information on Latens.no easy to read. Vamos et al [33] explored women’s experiences of accessing, understanding, appraising, and applying health information during pregnancy; they found that “women understand information best when visuals, statistics, tailored message and plain language is used.” This may explain our findings and indicate that the efforts made in developing the website were effective: the use of custom-made illustrations, user involvement from the beginning to help tailor the message, and the SAM to ensure the use of plain language with appropriate writing style and sentence construction. Nevertheless, the content on Latens.no was written by health personnel, and previous research indicates that there may be a discrepancy between how providers and patients perceive language use on websites [34]. As such, it was reassuring that the target users found it easy to read.

Although we had refined the design of Latens.no using the SAM [21], our opinion on the visual appearance was that it was quite simplistic; as such, we expected to receive input on this aspect of the design. In contrast to our expectations, however, few participants received negative feedback regarding the quality of the design. Instead, the findings show overall positive feedback on design, and our participants found Latens.no to be easy to navigate, with an easy-to-use and clear structure. This might be a consequence of the SAM refinement, in which graphic illustrations, lists, layout, and typography were all tailored to best *fit* the users. These results are in line with previous studies evaluating the feasibility and acceptability of mobile health lifestyle and medical apps to support health care during pregnancy in high-income countries [32]. In their systematic review, the authors found that mobile health technology is often judged to be good, easy, and simple to use [32].

Finally, despite the limited negative feedback, minor adjustments made as a result of this study resulted in a more professional-looking website.

### Strengths and Limitations

We consider it a strength that both LGH and ML have conducted several similar studies [35,36]. One limitation of this study is that it was conducted with a small sample, as is typical of qualitative studies [22]. We chose to apply the think-aloud method at the end of the qualitative interviews, as this is a widely used and accepted method of user involvement [37]. Latens.no is intended to function as a source of information not only in pregnancy but also during early labor; therefore, another limitation of this study is that we did not explore how our participants experienced Latens.no when in early labor. However, this was intended, as we did not want to impose a burden on our participants. Moreover, even though women’s partners were recruited alongside the women, there were more women than men in the sample. However, all participants gave information-rich and detailed descriptions of their experiences with Latens.no, and we see our inclusion of even a limited number of partners as a strength. Previous research on internet use in pregnancy has focused primarily on pregnant women [29,30,32]. However, the partner is potentially an important resource in pregnancy and birth and should be included to a greater extent in further research. A final limitation is that non-Norwegian-speaking women were excluded from the study because Latens.no is currently available only in Norwegian.

### Conclusions

The think-aloud method while performing tasks allowed for detailed feedback. The participants both confirmed the user-friendliness of the website but at the same time provided information enabling improvement.

We expect that changes made based on this user-centered design study will further increase the usability and acceptability of Latens.no. This website’s influence on women’s experience of early labor and on several obstetric outcomes will be tested in a prospective *before and after* the intervention study.

## Appendix

Multimedia Appendix 1 Screenshot of the front page (alpha version).

Multimedia Appendix 2 Screenshot of the front page (alpha version), including menu and search function.

Multimedia Appendix 3 Interview guide.

Multimedia Appendix 4 Screenshot of the front page (final version).

Multimedia Appendix 5 Screenshot of the front page (final version), scrolled down.

Multimedia Appendix 6 Screenshot of the front page (final version), including menu and search function.

## Abbreviations

SAM: Suitability Assessment of Materials
