# Supplementary figures and images for: Development of and Experiences With an Informational Website on Early Labor: Qualitative User Involvement Study
Source: JMIR Form Res. 2021 Sep 27;5(9):e28698. doi: 10.2196/28698 (PMC8506263; doi:10.2196/28698)

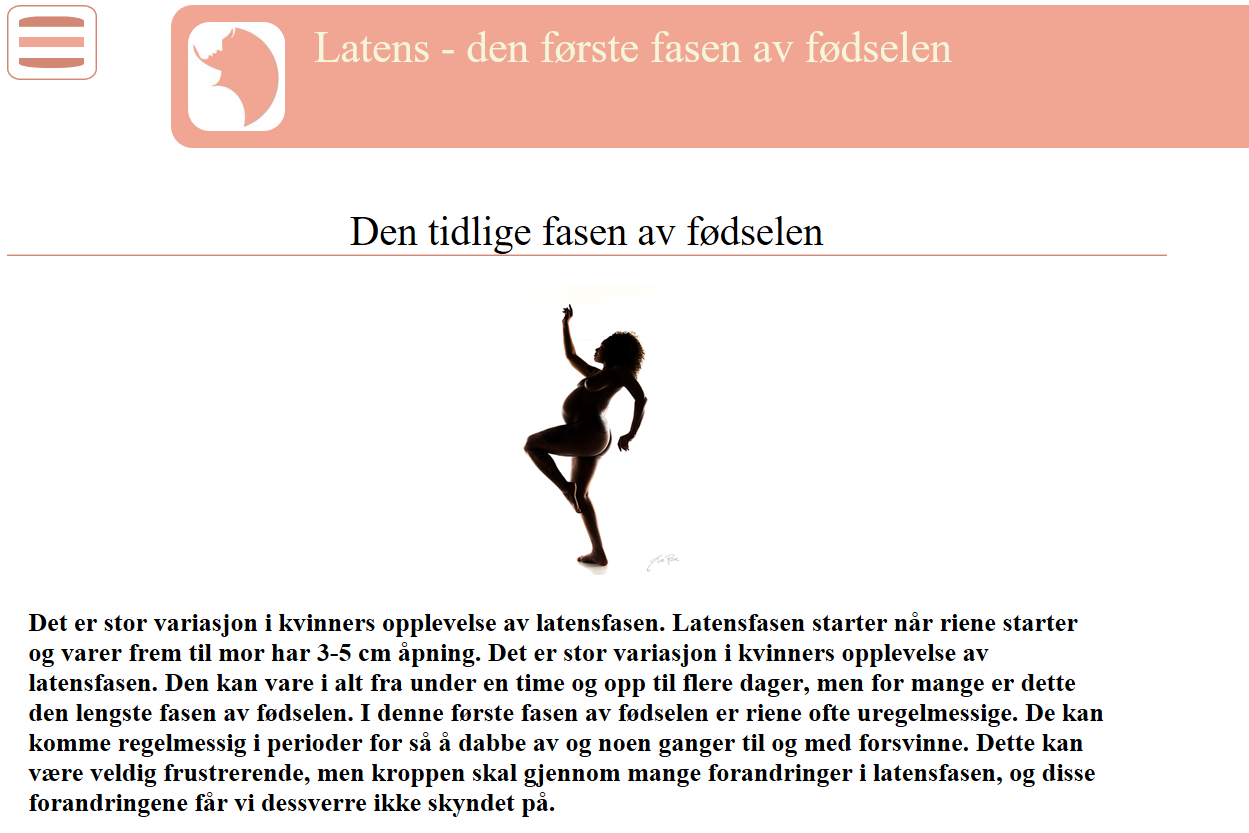

Supplement: Multimedia Appendix 1 [file formative_v5i9e28698_app1.png]

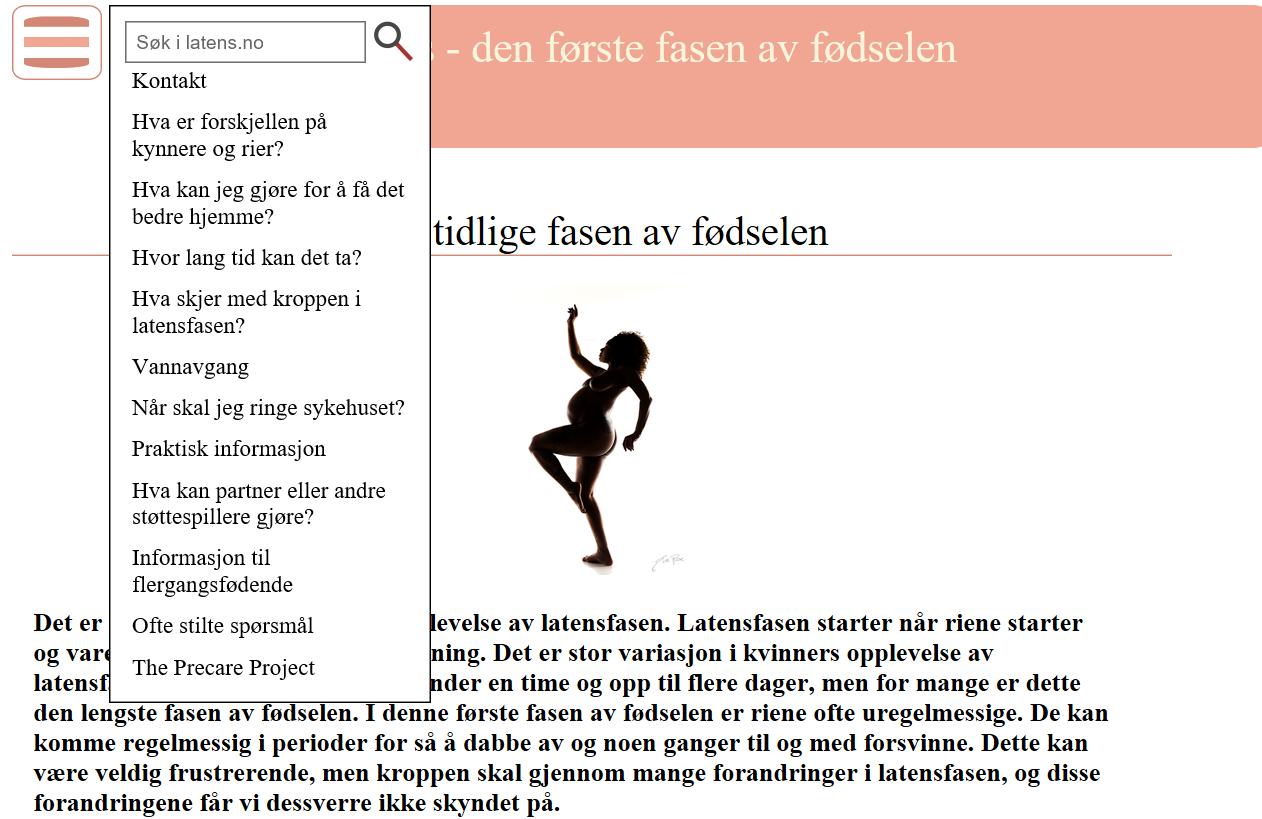

Supplement: Multimedia Appendix 2 [file formative_v5i9e28698_app2.png]

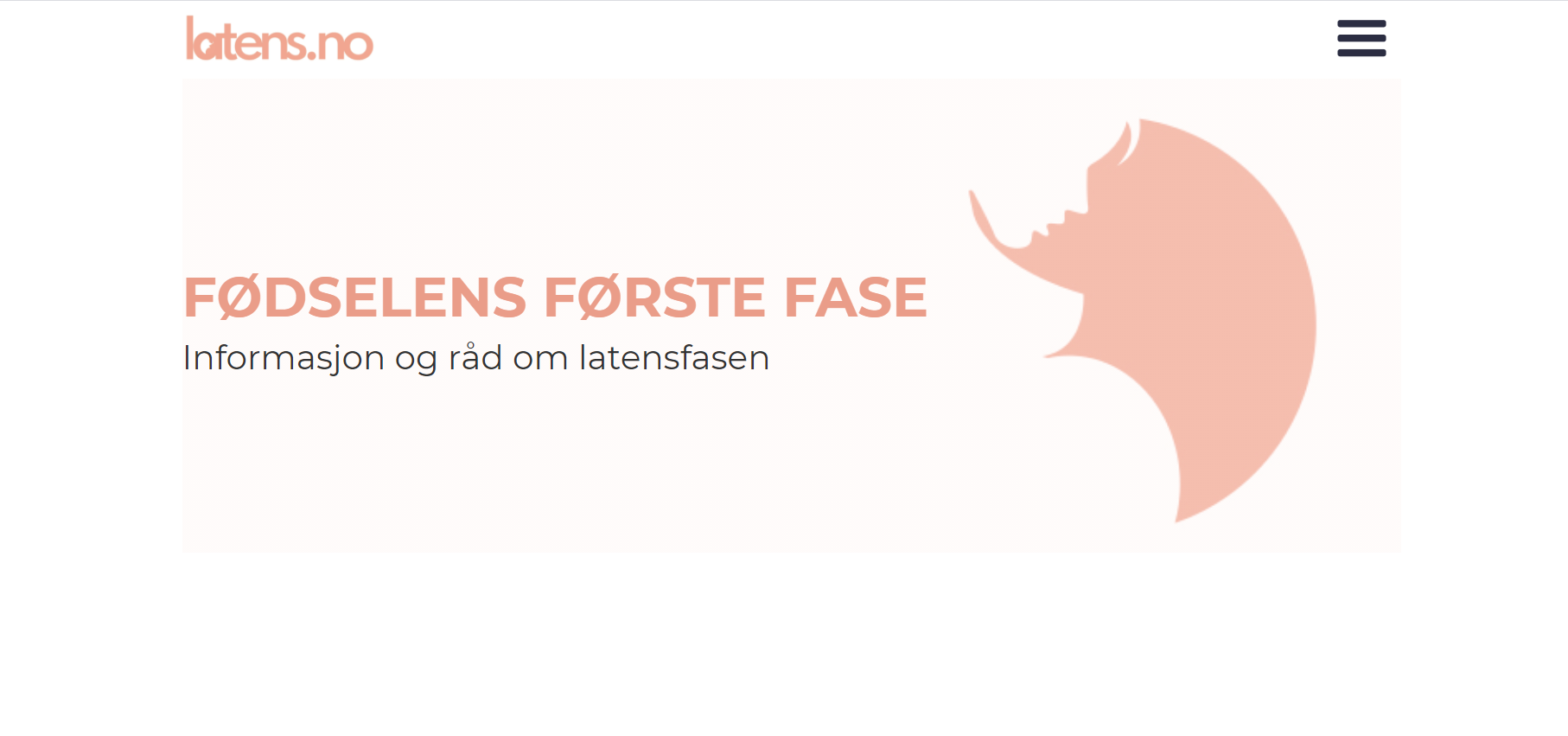

Supplement: Multimedia Appendix 4 [file formative_v5i9e28698_app4.png]

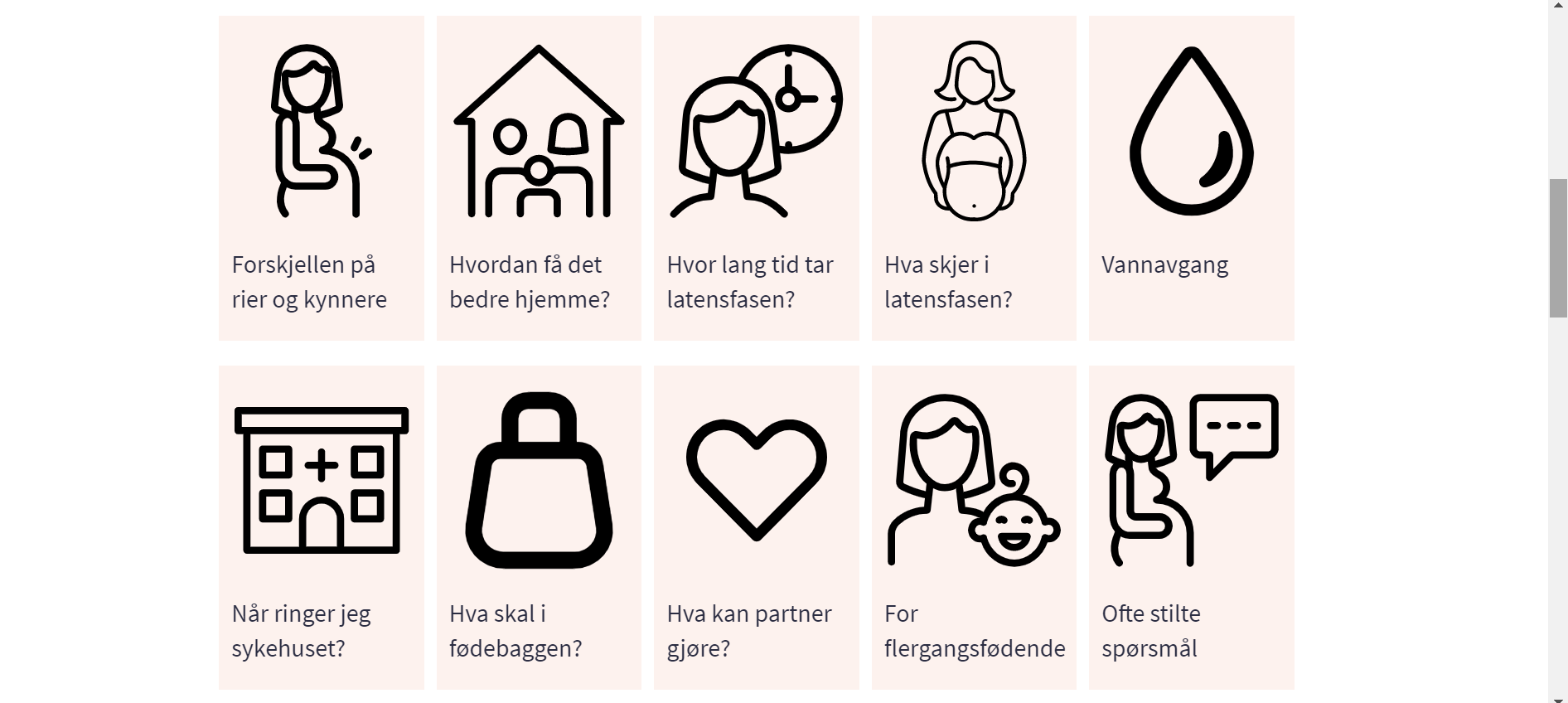

Supplement: Multimedia Appendix 5 [file formative_v5i9e28698_app5.png]

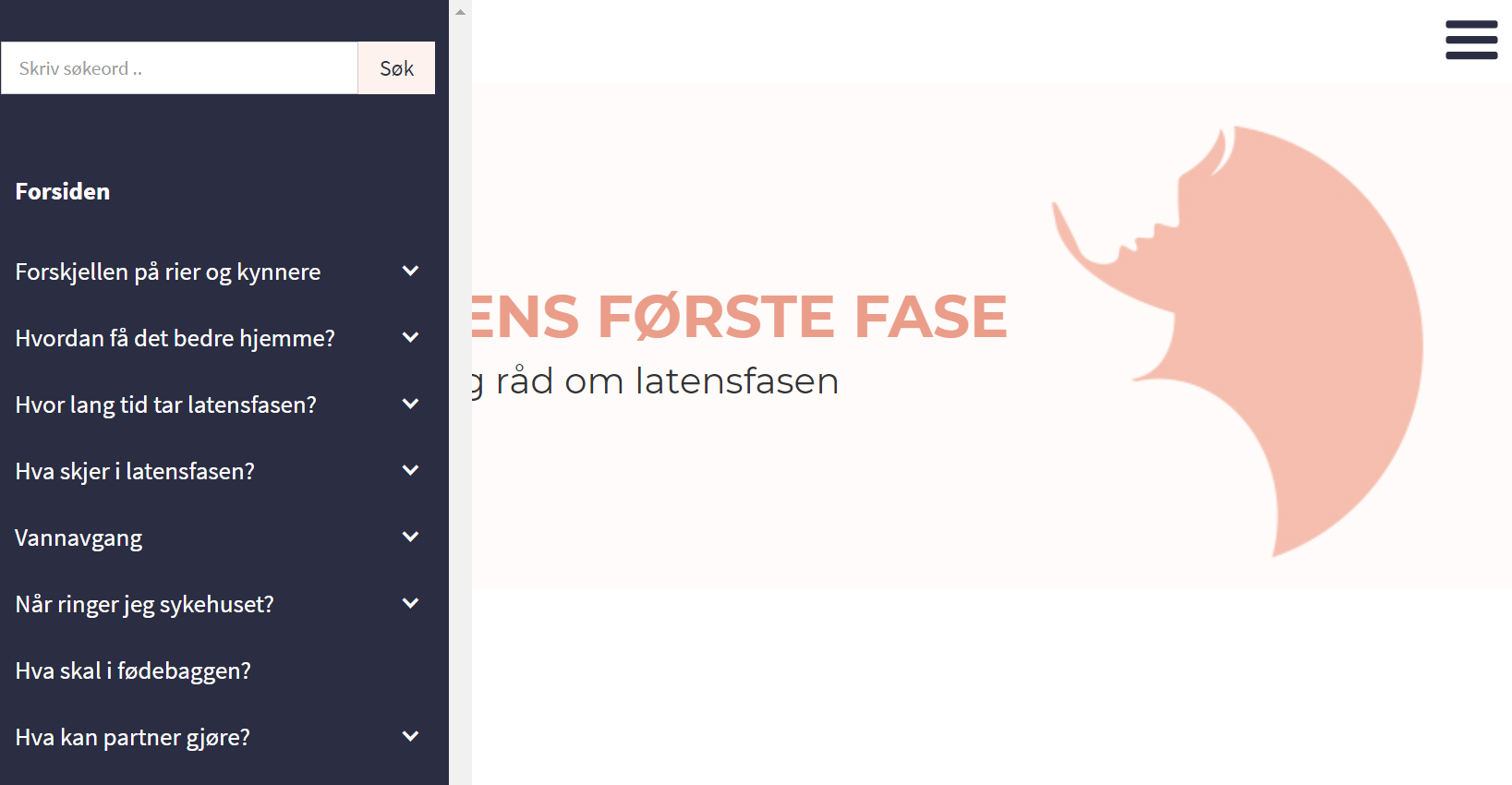

Supplement: Multimedia Appendix 6 [file formative_v5i9e28698_app6.png]
