# Supplementary material for: Development of and Experiences With an Informational Website on Early Labor: Qualitative User Involvement Study
Source: JMIR Form Res. 2021 Sep 27;5(9):e28698. doi: 10.2196/28698 (PMC8506263; doi:10.2196/28698)
Supplement: Multimedia Appendix 3 [file formative_v5i9e28698_app3.docx]

# Interview guide

**Background information:**

Age:

Age of child/pregnancy week:

Partner at home during early labor: Yes/no

Place of delivery/planned place of delivery:

Education:

Ethnicity/born in Norway:

Performed the testing on: Mobile phone/tablet/PC

**Questions to be asked prior to the introduction of the website:**

1. To the informants who had given birth: Do you have any experience with using a website in early labor?

To the informants who had not yet given birth: Do you have any experience with using a website to find information on pregnancy and birth?

– If yes: What made a good website good, and what made a poor website poor?

1. In your opinion, what should a website on early labor contain?
2. How do you think it should be designed?

**Tasks to be performed on the website using the “think aloud” method:**

*We want to find out if it is easy to find information related to early labor. A few hypothetical situations will now be described. I want you to find solutions for the situations using the website. I want you to think aloud while performing the tasks. Say what you are thinking, what you are doing, what you are looking at, and what you are feeling.*

1. At irregular intervals, you feel that your belly is painful and tight. But you are not sure if it is contractions or Braxton Hicks contractions. Can you find any information on the website that can help you assess this?
2. You have had contractions for a while, and then you discover spots of blood on the toilet paper after you use the toilet. Is this normal?
3. You are having contractions, but they are not painful enough for you to consider calling the hospital. Then your water breaks. Can you find out what you should do next?
4. You wonder what you should eat when you are at home with contractions. Can you find any recommendations on the website?
5. You want to get in touch with those responsible for the website to give feedback. Can you find the contact information?
6. Can you find out what it is wise to pack for the hospital?
7. You cannot seem to find a position that is comfortable when you are having contractions. Can you find something on the website that gives advice on whether you should stand or lie down?
8. You feel that you have had contractions for a very long time, but when you call the hospital, you are told to stay home a little longer. Can you find out if it is normal to have contractions for as long as you have?
9. When you call the hospital, the midwife talks about your “cervix.” What is the cervix?
10. How can your partner/you contribute when at home in early labor?

**Questions afterwards:**

1. How did you experience finding the information?
2. What do you think about the design of the website?
3. What do you think about the way the information is presented?
4. What do you think about the use of images? Should there be more pictures? What type of pictures? Illustrations or real photographs?
